# Application of Ginsenoside Rd in Periodontitis With Inhibitory Effects on Pathogenicity, Inflammation, and Bone Resorption

**DOI:** 10.3389/fcimb.2022.813953

**Published:** 2022-04-11

**Authors:** Shuhui Zhou, Yaoting Ji, Hantao Yao, Haiying Guo, Zichen Zhang, Zijun Wang, Minquan Du

**Affiliations:** The State Key Laboratory Breeding Base of Basic Science of Stomatology [Hubei-Ministry of Science and Technology of the People's Republic of China (MOST)] and Key Laboratory of Oral Biomedicine Engineering Ministry of Education, School and Hospital of Stomatology, Wuhan University, Wuhan, China

**Keywords:** ginsenoside, periodontitis, *Porphyromonas gingivalis*, inflammation, osteoclastogenesis

## Abstract

Periodontitis is a worldwide oral disease induced by the interaction of subgingival bacteria and host response and is characterized by local inflammation, bone resorption, and tooth loss. Ginsenoside Rd (Rd) is a biologically active component derived from Panax ginseng and has been demonstrated to exert antibacterial and anti-inflammatory activities. This study aims to investigate the inhibitory efficiency of Rd towards *Porphyromonas gingivalis* (*P. gingivalis*), periodontal inflammatory response, and osteoclastogenesis *in vitro* and to further validate the results in a mouse periodontitis model, thus, evaluate the potential effects of Rd on the control and prevention of periodontitis. According to the results, Rd exerted excellent antibacterial activities against planktonic *P. gingivalis*, along with attenuating *P. gingivalis* virulence and inhibiting its biofilms. Meanwhile, the inflammatory cytokine production and osteoclastogenesis were remarkably inhibited by Rd both *in vitro* and *in vivo*. Furthermore, Rd efficiently ameliorated the subgingival *P. gingivalis* abundance and suppressed the alveolar bone resorption *in vivo* as well. In conclusion, Rd has the potential to be developed as a promising medication in the control and prevention of periodontitis.

## Introduction

Periodontitis is one of the most common oral health problems, which is regarded as the major reason of tooth loss (Tonetti et al., 2017). *Porphyromonas gingivalis* (*P. gingivalis*) is a member of the subgingival red complex (Socransky et al., 1998), which plays an important role in the initiation and progression of periodontitis (Hajishengallis et al., 2012). Multiple studies have shown that *P. gingivalis* can invade and damage periodontal tissues benefiting from its gingipains and fimbriae (Atsuo, 2003; Sheets Shaun et al., 2005; Guo et al., 2010; Bostanci and Belibasakis, 2012); meanwhile, host responses such as local inflammatory responses and osteoclastogenesis are also indispensable for tissue destruction. Thus, to prevent and control periodontitis, it is important to simultaneously focus on the pathogenicity of *P. gingivalis*, as well as on tissue destruction resulting from host responses.

The current therapeutic strategy for periodontitis includes mechanical debridement, antibiotics, and periodontal surgery, which only targets removing bacteria, but the modulation towards host responses leading to tissue destruction is not involved. Moreover, present methods are sometimes limited due to the mechanical damage to periodontal tissues, antibiotic-resistant bacteria, and patients’ associated pain (Hasan and Palmer, 2014). In recent years, attention has been widely paid to edible natural herbs due to their high biological security and various pharmacological activities (Palombo, 2011; Kouidhi et al., 2015), which provide a new candidate for periodontal therapeutic strategy.

Ginsenosides, the main biological constituents of ginseng, which is a traditional Chinese herb with long medicinal history, have received considerable attention for their multiple bioactivities. According to their sapogenin, ginsenosides can be classified into protopanaxatriol, protopanaxadiol, and oleanolic acid ginsenosides, and it has been demonstrated that protopanaxadiol ginsenosides exert good properties in aspects of anti-inflammation, anti-microbism, neuroprotection, anti-neoplasm, anti-oxidation, reduction of plasma glucose, and so on (Lee et al., 2012; Hainan, 2006). In the stomatological field, the efficiency of ginsenosides has been mainly investigated towards oral pathogens (Trammell et al., 2012; Cao et al., 2019; Wang et al., 2020), and their effects on host modulation have been rarely discussed.

Based on the above information, our study aimed to investigate the biological properties of protopanaxadiol ginsenoside Rd (Rd) on the growth and virulence of *P. gingivalis*, as well as on the inflammation and bone resorption *in vitro* and *in vivo*, to assess the potential of Rd in the control and prevention of periodontitis.

## Materials and Methods

### Drug Preparations

Rd (Shanghai Yuanye Biotech, China) was dissolved in dimethyl sulfoxide (DMSO) to obtain a storage concentration of 100 mM and was diluted with culture mediums to final concentrations. The toxicity of DMSO towards *P. gingivalis* was determined beforehand.

### Bacterial Strain and Growth Condition

*P. gingivalis* (ATCC 33277) was cultured in ATCC medium 2722 (TSB medium), which was composed of 30 mg/ml trypticase soy broth (TSB, Becton, Dickinson and Company, USA), 5 μg/ml hemin, 1 μg/ml vitamin K_1_, 0.5 mg/ml l-cysteine hydrochloride, and 5 mg/ml yeast extract, at 37°C under anaerobic conditions (10% CO_2_, 10% H_2_, and 80% N_2_) in an anaerobic culture jar (Anoxomat Mark, the Netherlands). Blood agar plates were prepared by TSB mediums supplemented with 15 mg/ml agar and 5% sheep blood.

For each individual assay, *P. gingivalis* was cultured in TSB mediums, and the suspensions were standardized to an optical density (OD) of 1.0 at 600 nm using an ultraviolet spectrophotometer (SHIMADZU, Japan), which corresponded to 1.02×10^9^ colony-forming units (CFUs)/ml according to the preliminary determination.

### Cell Culture and Cell Viability Assay

Human gingival fibroblasts (HGFs) and RAW264.7 cells were purchased from the American Type Culture Collection (ATCC) and were respectively cultured in minimum essential medium α (α-MEM, Hyclone, USA) or Dulbecco’s modified Eagle’s medium (DMEM, Hyclone, USA) supplemented with 10% fetal bovine serum and 1% penicillin–streptomycin solution. For each individual assay, cells at passages 2–3 with 90% confluence were used.

Bone marrow-derived macrophages (BMDMs) were isolated from the femur marrow of mice. Specifically, bone marrows were flushed with α-MEMs and centrifuged to harvest pellets. Cells were then resuspended by α-MEM containing 10% FBS and incubated at 37°C for 24 h. Unattached cells were collected and treated with 50 ng/ml macrophage-colony-stimulating factor (M-CSF, R&D Systems, USA) for 48 h, and the adherent cells were BMDMs.

Cell viability was determined using the Cell Counting Kit-8 (CCK-8, Dojindo Laboratories, Japan). HGFs and RAW264.7 cells were seeded in 96-well plates at a density of 10^4^ cells/well, while the density of BMDMs was 10^5^ cells/well. After cell adherence, culture mediums were replaced by fresh mediums containing different concentrations of Rd (50, 100, 150, and 200 μM) and cells were incubated for 24 h. The CCK-8 reagent (10%, V/V) was then added to each well and incubated at 37°C for 45–60 min in the dark, followed by OD measurements at 450 nm. All sets were conducted in triplicate.

### Minimum Inhibitory Concentration and the Minimum Bactericidal Concentration of Rd

The minimum inhibitory concentration (MIC) was defined as the lowest drug concentration that the tested well showed the same turbidity as the blank control. Also, the minimum bactericidal concentration (MBC) was defined as the lowest drug concentration that had no bacteria growing on the tested blood agar plates.

Rd solution was serially diluted twofold in TSB medium to obtain a range of final concentrations (800–50 μM), then *P. gingivalis* (1 × 10^8^ CFU/ml) was treated with Rd in 96-well plates. The TSB medium without drug and bacteria served as the blank control, and the bacteria suspension without drug served as the normal control. The plates were incubated at 37°C for 24 h under anaerobic conditions, and OD values were measured at 600 nm by a microplate reader (BioTek Instruments, USA). After that, 5 μl of samples from each well that showed the same clarity as the blank control were inoculated on blood agar plates and incubated at 37°C for 7 days under anaerobic conditions. All sets were conducted in triplicate.

### Short-Term Inhibitory Effects on *P. gingivalis*

To determine the short-term antibacterial effects of Rd, *P. gingivalis* (1 × 10^6^ CFU/ml) was treated with Rd (1/8×, 1/4×, and 1/2× MIC), followed by continuous incubation for 12 h. At intervals of 3, 6, and 12 h, samples from each group were diluted 100-fold and then inoculated on blood agar plates and incubated at 37°C for 7 days under anaerobic conditions before CFUs were counted. All sets were conducted in triplicate.

### Scanning Electron Microscopy Analysis

*P. gingivalis* was treated with Rd (1/4× and ½× MIC) in a 48-well plate with the presence of coverslips for 24 h. Coverslips with adhering bacteria were washed with phosphate-buffered saline (PBS) and fixed with 400 μl 4% glutaraldehyde overnight. Then, the dehydration was performed using 75%, 80%, 90%, 95%, and 100% ethanol in turns, and samples were kept in 100% ethanol until the drying process. After that, critical point drying and metal spraying were conducted. Samples were scanned and pictured at 5000× and 20,000× magnification by scanning electron microscopy (SEM; Sigma, Zeiss AG, Germany).

### Crystal Violet Assay

A period of 48 h was used for treatment to form stable biofilms of *P. gingivalis*. Bacteria were treated with Rd (800–50 μM) for 48 h in a 96-well plate. After incubation, the culture medium with planktonic bacteria was discarded and the wells were washed with sterile water, followed by drying at room temperature. Then, 50 μl of 0.1% crystal violet dye (Shanghai Hushi, China) was added and kept for 15 min. After washing away the unstained dye, 100 μl of 30% acetic acid was used to solubilize the stained dye, and OD values were measured at 562 nm by a microplate reader. All sets were conducted in triplicate.

The half maximal biofilm inhibitory concentration (MBIC_50_) was defined as the lowest concentration of Rd that reduced the biomass of biofilms by at least 50% (Zhong et al., 2019).

### Cell-Surface Hydrophobicity of *P. gingivalis*

Cell-surface hydrophobicity (CSH) was evaluated using the microbial adhesion to hydrocarbons (MATH) method (Shafiei et al., 2020), which spectrophotometrically determines the percentage of bacterial cells in the aqueous suspension migrating to the hydrophobic hydrocarbon phase. Specifically, *P. gingivalis* (2 × 10^9^ CFU/ml) was treated with Rd (1/4× and ½× MIC) for 6 and 12 h at room temperature, and the cultures were transferred to sterile tubes. Bacterial cells were harvested by centrifugation at 6,000 rpm for 10 min at 4°C and were resuspended with 400 μl of phosphate urea magnesium (PUM) buffer (pH = 7.1). OD values were then measured at 500 nm (A0) by an ultraviolet spectrophotometer. Next, 200 μl of *n*-hexadecane (Shanghai Aladdin Biotech, China) was added and mixed uniformly for 60 s by a vortex mixer. After that, mixtures were stood for 15 min to separate the phases, and OD values of the aqueous phase was measured at 500 nm.

CSH was represented as the hydrophobic index: [(A0−A1)/A0] × 100%. All sets were conducted in triplicate.

### Growth Curves of Biofilms

To determine the bacterial population and viability retained in the biofilm upon Rd treatments, the growth curves of biofilms were plotted. *P. gingivalis* and Rd (1/2× and 1× MBIC_50_) were cocultured in a 48-well plate with the presence of coverslips for 48 h. Biofilms growing on coverslips were transferred into 5 ml TSB mediums and continuously incubated for 70 h, and OD values at 600 nm were measured at a series of time points (20, 30, 40, 45, 50, 65, 70, and 75 h). All sets were conducted in triplicate.

### Confocal Laser Scanning Microscopy Analysis

*P. gingivalis* was treated with Rd (1/2× and 1× MBIC_50_) in a 24-well plate with the presence of coverslips for 48 h. Biofilms growing on coverslips were stained with a LIVE/DEAD BacLight Bacterial Viability Kit (ThermoFisher, USA). The serial layer scanning was performed at intervals of 2 μm, and 3D images of confocal laser scanning microscopy (CLSM; Leica Biosystems Nussloch GmbH, Germany) were reconstructed with the Leica Application Suite X (LAS X) software.

### Enzyme-Linked Immunosorbent Assay

An inflammatory model was established by 1 μg/ml of lipopolysaccharide (LPS) (InvivoGen, China) that originated from *P. gingivalis*. Concretely, HGFs were pretreated with Rd (0 and 100 μM) for 3 h, followed by LPS induction for 24 h. The supernatants were then collected, and concentrations of interleukin (IL)-6, IL-8, and IL-1β cytokines were determined using enzyme-linked immunosorbent assay (ELISA) kits (NeoBioscience, China) according to the ‘manufacturer’s protocols. All sets were conducted in triplicate.

### Osteoclastogenesis Induction

RAW264.7 cells and BMDMs were pretreated with Rd (0, 50, and 100 μM) and were then treated with a receptor activator of nuclear factor-κB ligand (RANKL, 50 ng/ml, R&D Systems, USA) for 6 days. The group without Rd and RANKL treatments served as the control. After that, cells were fixed and the tartrate-resistant acid phosphatase (TRAP) staining was performed using a leukocyte acid phosphatase kit (Sigma-Aldrich, USA). TRAP-positive (+) multinucleated (nuclei ≥3) cells were counted under light microscopic observation (Nikon, Japan). All sets were conducted in triplicate.

### Total RNA Isolation and Quantitative Reverse Transcription-Polymerase Chain Reaction Analysis

Quantitative reverse transcription-polymerase chain reaction (qRT-PCR) assay was used to determine expressions of virulent genes in *P. gingivalis* (*fimA* and *kgp*), inflammatory cytokine genes (*IL1B*, *IL6*, and *CXCL8*), and osteoclast marker genes (*Acp5*, *Nfatc1*, and *Mmp9*), along with the relative quantity of *P. gingivalis* in mice gingival crevicular fluids.

Total RNA was extracted using RNAiso reagent (Takara Biotech, China) according to the manufacturer’s protocol. RNA concentrations and purity were determined by Nanodrop 2000 (ThermoFisher, USA). RNA was converted to cDNA with HiScript II Q RT SuperMix (Vazyme Biotech, China).

Gene-specific primers (**Table 1**) were designed by Sangon Biotech Company (China) and checked by primer BLAST (National Center for Biotechnology Information, USA). Reaction mixtures consisted of 2× ChamQ SYBR qPCR Master Mix (Vazyme Biotech, China), cDNA template, and primers. The reaction was set according to the manufacturer’s protocol: an initial denaturation for 30 s at 95°C, followed by a 40-time thermal cycle of 95°C for 10 s and 60°C for 30 s. The melting curve was collected using the built-in program of Bio-Rad, CFX96 (Bio-Rad Laboratories, USA). The *P. gingivalis 16S rRNA* gene, the mouse *GAPDH* endogenous gene, and the human *ACTB* endogenous gene served as the reference genes, respectively, and relative levels of gene expression were normalized by the comparative Ct method (2^−ΔΔCt^ method). All sets were conducted in triplicate.

**Table 1:** Primers used for qRT-PCR.

Primer	Sequence	Product size (bp)
*P. gingivalis 16S rRNA*	Forward 5’-GGTGCGTAGGTTGTTCGGTAAGTC-3’	95
	Reverse 5’-CTGCCGCCGCTGAACTCAAG-3’	
*kgp*	Forward 5’-ACCTACACTCAAGGAGGAGCCAAC-3’	146
	Reverse 5’-GGACCTTCGCCTTCACCTGTTATC-3’	
*fimA*	Forward 5’-TCTTGTTGGGACTTGCTGCTCTTG-3’	90
	Reverse 5’-CGCTGATGGTGGCATTACCTTCTG-3’	
*IL1B*	Forward 5’-ATGATGGCTTATTACAGTGGCAA-3’	132
	Reverse 5’-GTCGGAGATTCGTAGCTGGA-3’	
*IL6*	Forward 5’-CACTGGTCTTTTGGAGTTTGAG-3’	101
	Reverse 5’-GGACTTTTGTACTCATCTGCAC-3’	
*CXCL8*	Forward 5’-ACTGAGAGTGATTGAGAGTGGAC-3’	112
	Reverse 5’-AACCCTCTGCACCCAGTTTTC-3’	
*Acp5*	Forward 5’-CAAGAACTTGCGACCATTGTTA-3’	191
	Reverse 5’-ATCCATAGTGAAACCGCAAGTA-3’	
*Nfatc1*	Forward 5’-TCTCCTCCTTTCTGCCCACCTTC-3’	106
	Reverse 5’-GCCTTCTCCGATTGCTGTCATCC-3’	
*Mmp9*	Forward 5’-CAAAGACCTGAAAACCTCCAAC-3’	105
	Reverse 5’-GACTGCTTCTCTCCCATCATC-3’	
human *ACTB*	Forward 5’-ATTGCCGACAGGATGCAGA-3’	89
	Reverse 5’-GAGTACTTGCGCTCAGGAGGA-3’	
mouse *GAPDH*	Forward 5’-CCGCCTGGAGAAACCTGTATGTATG-3’	140
	Reverse 5’-ATGCCTGCTTCACCACCTTCTTG-3’	

### Establishment of a Mouse Periodontitis Model

The animal experiment protocol was approved by the Animal Ethics Committee for Experimental Research of Wuhan University (permission number: S07921040I) and conducted following the committee’s guidelines.

Male-specific pathogen-free C57BL/6 mice (9 weeks old) were randomly allocated to three groups (*n* = 8 per group): control, periodontitis, and Rd. The periodontitis model was established according to previous studies (Li et al., 2018; Sun et al., 2020), with slight modifications (**Figure 5A**). Briefly, a 3-day oral penicillin treatment was given to each group. Next, the experimental periodontitis was modelled by ligaturing sterile sutures around the second maxillary molar under intraperitoneal anesthesia on the second day. The control group also accepted the anesthesia as a sham operation. Bacterial suspensions (2 × 10^9^ CFU/ml), containing 2% carboxymethyl cellulose, were then smeared surrounding the ligatured teeth once a day. Rd (300 μM in PBS) treatments were conducted by local injection in the Rd group every other day since the ligation. Sterile PBS was used as a placebo instead of Rd solutions in the periodontitis group. The drug or placebo needed to be injected into the gingival sulci. The control group received sterile PBS only. After the 8-day modelling, mice were euthanized with carbon dioxide. The unilateral maxillary alveolar bones were dissected and divided into halves, one-half for the histological analysis, and the other was used for morphometric and microcomputed tomography (micro-CT) analysis.

### *P. gingivalis* Abundance in Mice Gingival Crevicular Fluids

Gingival crevicular fluid (GCF) samples were collected by putting a #20 absorbent paper point (GAPADENT, China) into the buccal and palatal gingival sulci of the ligatured teeth for 5 s, respectively. Paper points were immersed in 500 μl of sterile PBS as soon as possible. After immersion at 4°C overnight to elute bacteria samples, 5 μl of samples were then inoculated on blood agar plates. Furthermore, *P. gingivalis* abundance was detected by qRT-PCR assay using the remaining samples, regarding the *P. gingivalis 16S rRNA* as the target gene, and values were normalized using a universal bacteria primers pair (Mazzoli et al., 2020).

### Morphometric and Micro-CT Analysis for Alveolar Bones

Alveolar bone samples were separated from soft tissues and stained with 0.5% methylene blue. Images were taken from both buccal and palatal sides using a stereomicroscope. The samples were then scanned using a micro-CT system (Skyscan 1276, Bruker, Germany), and the parameters were as follows: pixel size, 3.033835 μm; voltage, 55 kV; and electrical current, 200 μA. The 3-dimensional reconstruction was conducted by the Skyscan Ctvox software, and the distance from the cementoenamel junction (CEJ) to the alveolar bone crest (ABC) was measured both in the digital images and reconstructed images.

The region of interest (ROI) was defined as a trapeziform region around the roots of the secondary molar, and bone histomorphometry indexes [percent bone volume (BV/TV); bone surface density (BS/TV); and trabecular number (Tb.N)] in the ROI were evaluated using the Skyscan CTAn software.

### Histological Analysis for Periodontal Tissues

Alveolar bone samples with intact soft tissues were fixed in 4% paraformaldehyde for 48 h, decalcified with 10% ethylene diamine tetraacetic acid (EDTA) solution for 25 days, and embedded with paraffin. Tissue sections were prepared, followed by hematoxylin–eosin (HE) staining, TRAP staining, and immunohistochemical staining. Positive rates of IL-1β and IL-6 in immunohistochemically stained sections were acquired by calculating percentages of the brown-stained area.

### Statistical Analysis

All data presented are obtained from three independent experiments. The data were presented as mean ± standard deviation (SD). Significant differences were determined by Student’s *t*-test between two groups, and one-way analysis of variance (one-way ANOVA) was used for more than two groups in Prism 8.0 (GraphPad Software, USA). *p* < 0.05 was considered statistically significant.

## Results

### Inhibitory Effects of Rd on the Growth and Virulence of Planktonic *P. gingivalis*

**Figure 1:**
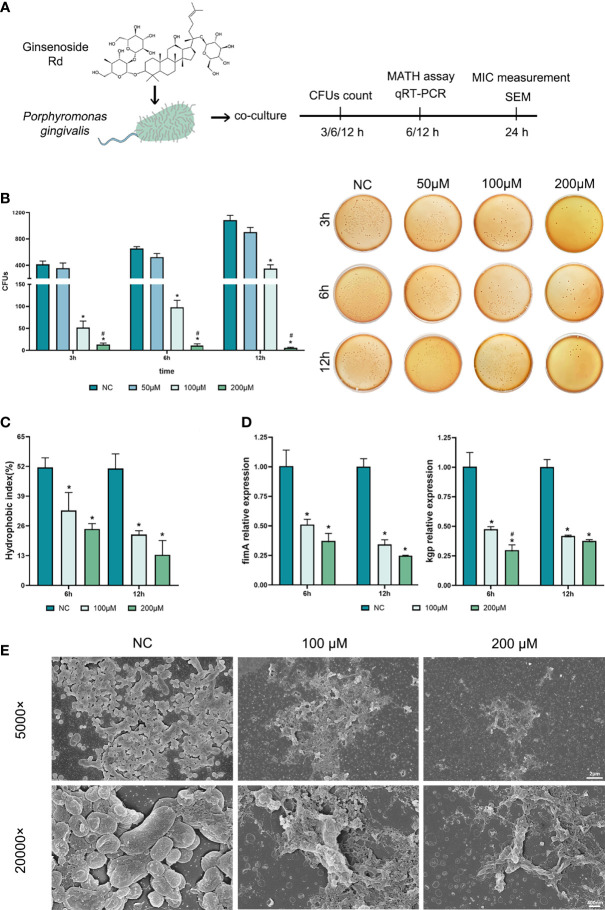
Antibacterial effects of ginsenoside Rd towards planktonic *P. gingivalis*. **(A)** The sketch of antibacteria experiments. **(B)** Short-term effects on *P. gingivalis* and representative photographs of agar plates were displayed. **(C)** The hydrophobic indexes of *P. gingivalis*. **(D)** Expressions of kgp and fimA genes were normalized by the 2^−ΔΔCt^ method relative to the *P. gingivalis* 16s rRNA gene. The results are shown as mean ± SD (*n* = 3). ^*^ *p* < 0.05 versus the normal control; ^#^ *p* < 0.05 versus the 100-μM group. **(E)** SEM images showed the population and morphology of *P. gingivalis* at magnifications of ×5,000 and ×20,000.

The basic antibacterial efficiency of Rd was detected by measuring the MIC and MBC, and we found that the MIC and MBC of Rd towards *P. gingivalis* were both 400 μM (**Table 2**). To evaluate inhibitory effects on bacterial growth and virulence concretely, different concentrations of Rd (50, 100, and 200 μμ) were used in subsequent assays. As shown in **Figure 1**, 3-h treatments of 200 and 100-μM Rd significantly reduced the amount of bacterial colonies in a dose-dependent manner (**Figure 1B**), and 200-μM Rd had also inhibited bacterial growth continuously during a 12-h period, while there was no significant difference between the 50-μM group and the control. The SEM images (**Figure 1F**) visually showed the decrease of bacterial quantity, as well as the destruction of cellular morphology. Untreated bacteria cells showed regular short bacilliform shapes and smooth surfaces, in contrast, cells exhibited obvious shrinkage and rupture after Rd (200 and 100 μM) treatments. To get rid of the interference of the resolvent, inhibitory effects of DMSO towards *P. gingivalis* were measured (**Figure S1**), and the result indicated that the highest dose of DMSO (0.4%) used in our study was nontoxic towards *P. gingivalis.*

**Table 2:** The MIC and MBC of ginsenoside Rd.

	MIC	MBC
*P. gingivalis*	400 μM	400 μM

MIC, minimum inhibitory concentration; MBC, minimum bactericidal concentration.

To investigate the effects of Rd on *P. gingivalis* virulence, changes in several virulence factors were evaluated. Dramatic reductions were observed in the hydrophobic index (**Figure 1C**) in the Rd-treated groups, denoting that Rd could weaken the CSH of *P. gingivalis* to a great extent. Moreover, qRT-PCR results (**Figure 1D**) indicated that expressions of virulent genes (*kgp* and *fimA*) were significantly downregulated by Rd as well as according to our data.

In summary, a 400-μM Rd showed a significant bactericidal activity towards *P. gingivalis*, while 200- and 100-μM Rd exhibited good inhibitory effects on bacterial growth and virulence factors, whereas the suppression of *P. gingivalis* virulence did not show any dose dependency.

### Inhibitory Effects of Rd on *P. gingivalis* Biofilms

**Figure 2:**
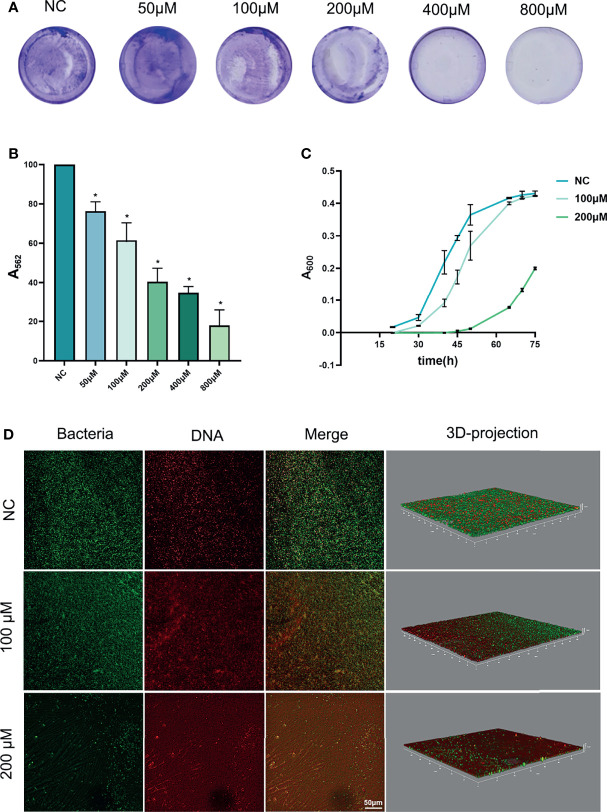
Antibiofilm effects of ginsenoside Rd towards *P. gingivalis* biofilms. **(A)** Photographs of biofilms stained by crystal violet, and **(B)** the biomass of biofilms was presented by the absorbance of dissolved stain. **(C)** Growth curves of biofilms. The results are shown as mean ± SD (*n* = 3). ^*^ *p* < 0.05 versus the normal control. **(D)** CLSM images revealed live or dead bacteria in biofilms.

Anti-biofilm effects of Rd are shown in **Figure 2**. The total biomass of biofilms, including living bacteria, dead bacteria, and extracellular matrices (Li et al., 2003) was measured by crystal violet assay (**Figures 2A, B** **)**. According to the results, the half maximal biofilm inhibitory concentration (MBIC_50_) of Rd was 200 μM, and the inhibitory effects of 100- and 50-μM Rd also showed statistical difference (*p* < 0.05).

Next, the growth curves of *P. gingivalis* biofilms (**Figure 2C**) indicated that 200-μM Rd exhibited a growth-inhibiting property towards surviving bacteria in treated biofilms over a period of 45 h, but the effect could not be maintained permanently.

Furthermore, the living and dead bacteria staining was conducted to reveal the bactericidal effects of Rd on *P. gingivalis* in biofilms. In CLSM images (**Figure 2D**), live cells showed a green fluorescence signal and the dead cells showed a red one. It could be visually found that bacteria were extensively killed in the 200-μM group, and the 100-μM Rd group also exerted a partial bactericidal ability in biofilms.

While excellent antibacterial and anti-biofilm effects were validated in *P. gingivalis*, the inhibitory effects of Rd on inflammation and osteoclastogenesis were subsequently investigated *in vitro*.

### Inhibitory Effects of Rd on Periodontal Inflammatory Cytokines *In Vitro*

**Figure 3:**
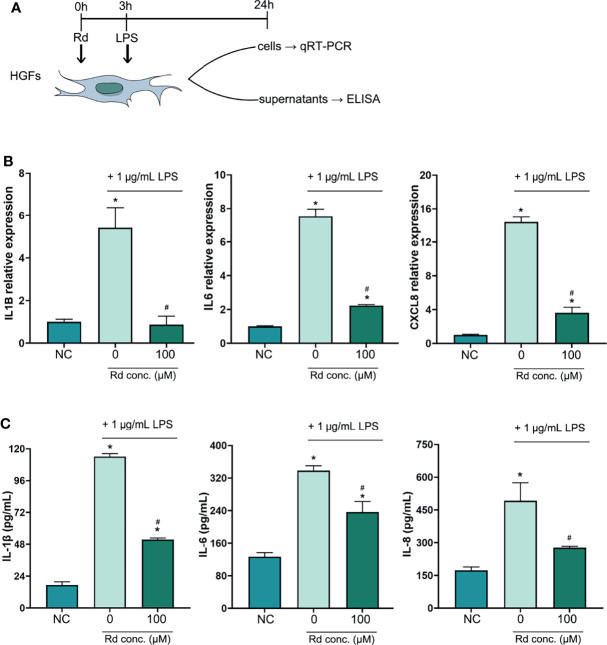
Inhibitory effects of ginsenoside Rd on inflammatory responses *in vitro*. **(A)** The sketch of anti-inflammation experiments *in vitro*. **(B)** Expressions of *IL1B*, *IL6*, and *CXCL8* genes were normalized by the 2^−ΔΔCt^ method relative to the human *ACTB* reference gene. **(C)** Concentrations of secreted IL-1β, IL-6, and IL-8 were determined by ELISA. The results are shown as mean ± SD (*n* = 3). ^*^ *p* < 0.05 versus the normal control; ^#^ *p* < 0.05 versus the 0-μM group.

An inflammation model was established in HGFs by LPS stimulation *in vitro*, and the CCK-8 assay (**Figure S2**) showed that the cell viability of HGFs was preserved for over 85% at a high Rd concentration (200 μM) and for 100% at concentrations below 150 μM, compared with the control. To evaluate the anti-inflammatory effects of Rd, a concentration of 100 μM was used and results are shown in **Figure 3**. qRT-PCR results (**Figure 3B**) showed that expressions of inflammatory genes (*IL1B*, *IL6*, and *CXCL8*) were elevated in LPS-stimulated HGFs and were obviously decreased by Rd compared with the LPS group. Similarly, ELISA results (**Figure 3C**) showed that Rd could antagonize the increase of secreted inflammatory cytokines (IL-1β, IL-6, and IL-8) in culture supernatants as well. Taken together, considerable anti-inflammatory effects of Rd were revealed in HGFs, as shown by the downregulation of inflammatory cytokines both in gene and secretion levels, while the effect on genes was more commendable.

### Inhibitory Effects of Rd on Osteoclastogenesis *In Vitro*

**Figure 4:**
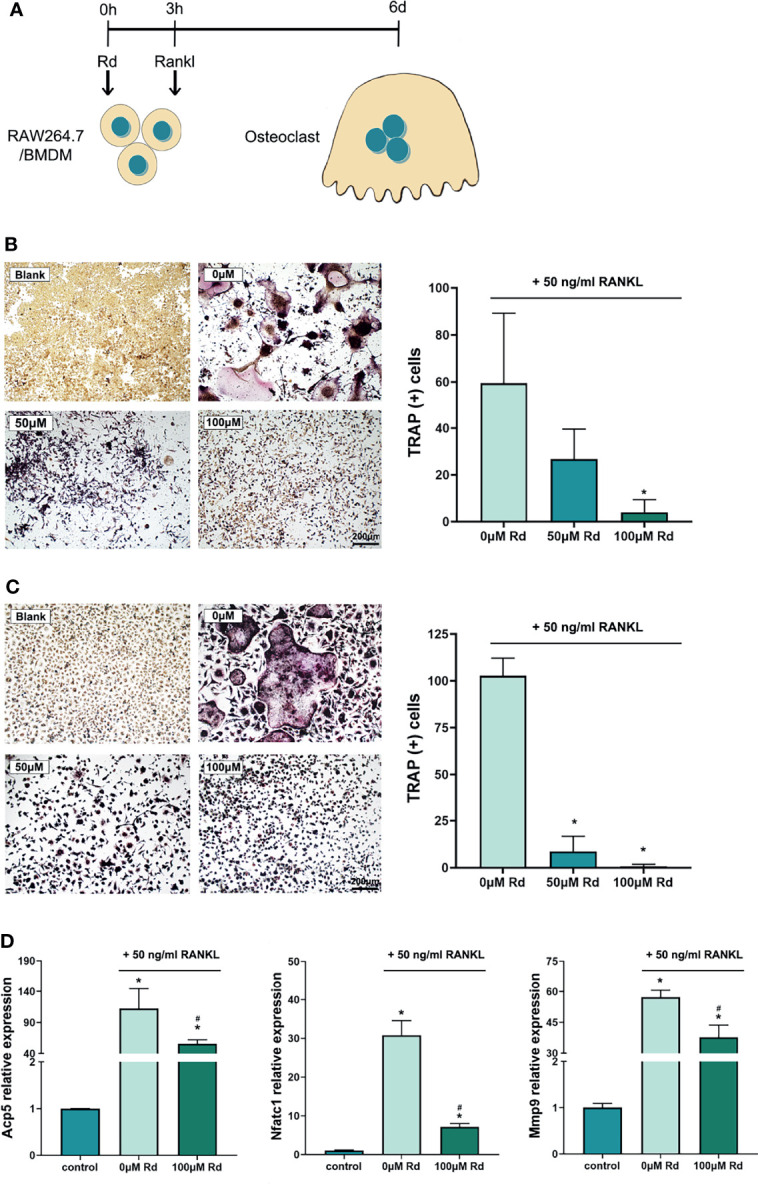
Inhibitory effects of ginsenoside Rd on osteoclastogenesis *in vitro*. **(A)** The sketch of antiosteoclastogenesis experiments *in vitro*. Photographs of multinucleated TRAP (+) cells differentiated from **(B)** RAW264.7 cells and **(C)** BMDMs were taken under ×10 magnification, and multinucleated cell amounts were counted. The results are shown as mean ± SD (*n* = 3). **(D)** Expression of osteoclast marker genes. The results were normalized by the 2^−ΔΔCt^ method relative to the mice *GAPDH* reference gene and shown as mean ± SD (*n* = 3). ^*^ *p* < 0.05 versus the normal control; ^#^ *p* < 0.05 versus the 0-μM group.

Osteoclastogenesis was experimentally induced by RANKL *in vitro*, and multinucleated osteoclasts were revealed by the TRAP staining. The CCK-8 assay (**Figure S2**) showed that the cell viability of RAW264.7 and BMDMs was preserved for over 90% and 100% at a high Rd concentration (200 μM), respectively, and for basically 100% at concentrations below 150 μM, compared with the control. As shown in **Figure 4**, treating with 100-μM Rd significantly reduced amounts of TRAP (+) cells both in RAW264.7 cells (**Figure 4B**) and BMDMs (**Figure 4C**), compared with the 0-μM group (*p* < 0.05), while a valid effect of 50-μM Rd was only observed in BMDMs. Furthermore, qRT-PCR results (**Figure 4D**) showed that expressions of osteoclast marker genes (*Acp5*, *Nfatc1*, and *Mmp9*) were upregulated under RANKL induction and could be effectively downregulated by 100-μM Rd in RAW264.7 cells, compared with the 0-μM group (*p* < 0.05). In summary, Rd played a great role in the inhibition of osteoclastogenesis *in vitro*, along with good retention of the cell viability.

### Inhibitory Effects of Rd on the Alveolar Bone Resorption and Destruction *In Vivo*

**Figure 5:**
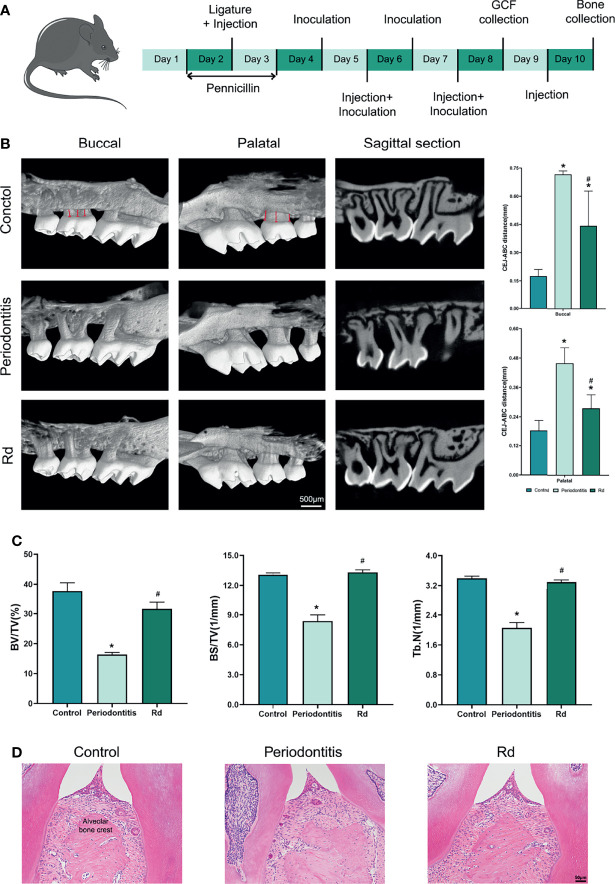
Ginsenoside Rd led the alleviation of bone resorption *in vivo*. **(A)** The sketch of the animal experiment. **(B)** Reconstructed images of the alveolar bones of mice were obtained by micro-CT scanning. The CEJ–ABC distance was indicated by the red lines and was measured according to the scale. **(C)** Bone histomorphometry indexes (BV/TV, BS/TV, and Tb.N) in the ROI. The results are shown as mean ± SD (*n* = 3). ^*^ *p* < 0.05 versus the normal control; ^#^ *p* < 0.05 versus the periodontitis group. **(D)** HE staining of the periodontal tissues of mice.

Taking the satisfactory *in vitro* results into account, a periodontitis model was established in mice and the effects of Rd on bone resorption and destruction were observed. The weight record and the HE staining for organs showed that Rd treatments did not exert biological toxicity in mice (**Figure S3**). To evaluate the alveolar bone resorption, CEJ–ABC distances were measured on digital (**Figure S4**) and reconstructed images (**Figure 5B**), respectively. The periodontitis group showed remarkable bone resorption compared with the control, while Rd treatments significantly lessened the CEJ–ABC distance compared with the periodontitis group (*p* < 0.05). On the other hand, HE staining also revealed obvious descents of the alveolar bone crest in the periodontitis group (**Figure 5D**), and the Rd group performed almost the same as the control, which further proved the inhibitory effect of Rd on the bone resorption. In addition, bone histomorphometry indexes (**Figure 5C**) were analyzed in a region of interest, and consistently, significant decreases were shown in the periodontitis group, while Rd treatments notably made improvements compared with the periodontitis group (*p* < 0.05). Therefore, Rd was verified to have inhibitory activities towards the alveolar bone resorption and destruction in experimental periodontitis.

### Effects on the Subgingival *P. gingivalis*, Inflammation, and Osteoclastogenesis *In Vivo*

**Figure 6:**
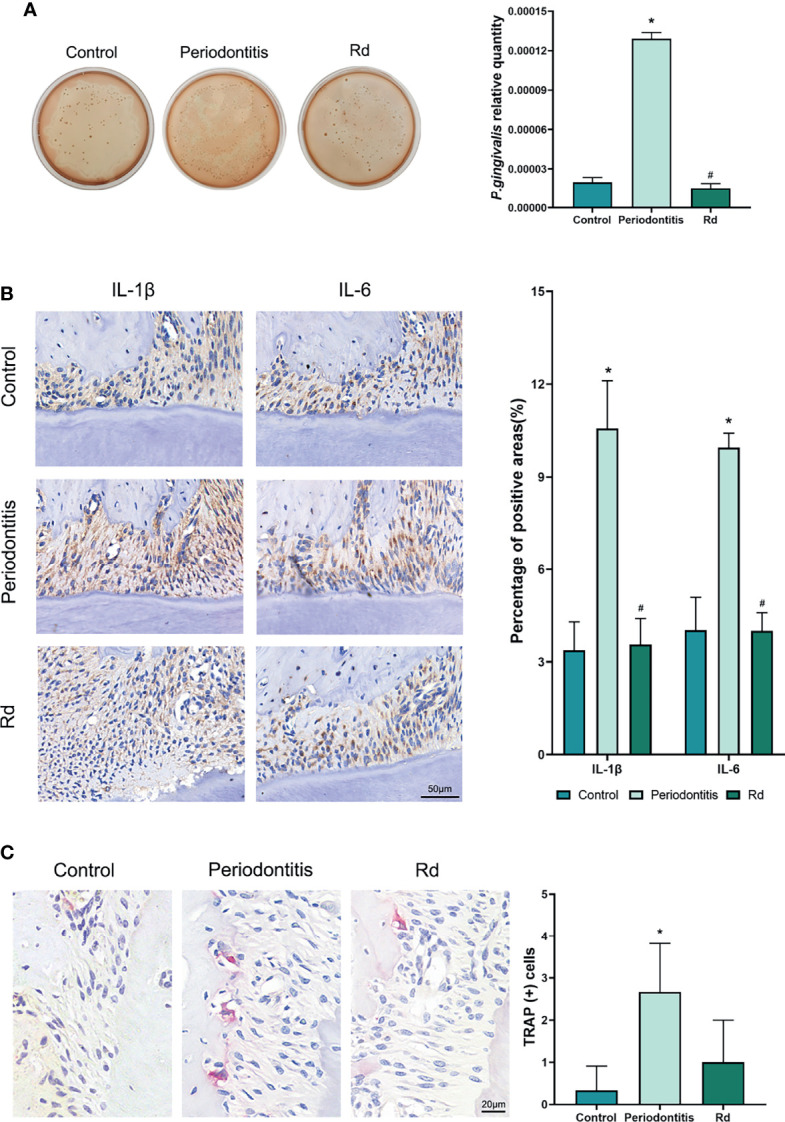
Effects of ginsenoside Rd on the *P. gingivalis* quantity, local inflammation, and osteoclastogenesis *in vivo*. **(A)** The total bacterial load in the GCF was shown by the plate spreading method, and the subgingival *P. gingivalis* abundance was quantitated by qRT-PCR. **(B)** Photographs of immunohistochemical staining and positive rates of IL-1β and IL-6 cytokines in tissue sections. **(C)** Photographs and counts for multinucleated TRAP (+) cells in tissue sections. The results are shown as mean ± SD (*n* = 3). ^*^ *p* < 0.05 versus the normal control; ^#^ *p* < 0.05 versus the periodontitis group.

Besides the bone mass, bacterial load and local inflammation, as well as osteoclastogenesis, were also detected *in vivo*. Blood agar plates (**Figure 6A**), which were inoculated with mice GCF samples, visually showed decreased bacteria colonies in the Rd group, compared with the periodontitis group. Considering the existence of miscellaneous bacteria that were still alive after the penicillin pretreatment (was shown by plates of the control group), the quantity of *P. gingivalis* related to total bacteria was measured by qRT-PCR, so as to represent the *P. gingivalis* abundance in GCFs. Results indicated that the relative quantity of *P. gingivalis* was significantly decreased by Rd treatment, compared with the periodontitis group (*p* < 0.05).

In tissue sections, immunohistochemical staining (**Figure 6B**) was conducted to reveal inflammatory cytokines (IL-1β and IL-6) in the periodontal tissues of mice, which showed significant reductions of positive rates under Rd treatments, compared with the periodontitis group (*p* < 0.05). Moreover, TRAP staining (**Figure 6C**) visually showed a reduction of TRAP (+) cells in the periodontal tissues of mice near the bone surface, whereas the difference in cell count between the periodontitis and Rd group was not significant (*p* > 0.05).

## Discussion

Periodontitis is a widespread oral disease that is characterized by periodontal inflammation, alveolar bone resorption, and tooth loss. It is currently considered that the dysbiosis between the periodontal microorganism and host defense leads to the occurrence of the disease (Page and Kornman, 1997): subgingival bacteria were the initial event of periodontitis, subsequently, virulence factors from bacteria and reactive cytokines from host responses jointly mediated the tissue destruction. It suggests that a combined strategy targeting pathogenic bacteria and host responses is likely to be more effective towards periodontitis, compared with traditional single antibacterial approaches. In this study, inhibitory properties of ginsenoside Rd were investigated in aspects of periodontal pathogen, inflammation, and bone resorption, thus, evaluated its applicable value in the control and prevention of periodontitis.

*P. gingivalis* is an oral commensal bacterium which can opportunistically mediate the initiation of periodontitis as a dominant pathogen. It is acknowledged that *P. gingivalis* not only processes intrinsic virulence towards periodontal tissue directly but also participates in promoting periodontal inflammation and alveolar bone loss (Maekawa et al., 2014; Ahmad et al., 2020). As other research reported (Xue et al., 2020), the antibacterial effect of Rd on *P. gingivalis* was unsatisfactory, which was only assessed by the MBC. Although an absolute bactericidal effect was similarly exhibited by a high Rd concentration of 400 μM in our study, thorough killing for an oral commensal bacterium was unprofitable, thereby more detailed research was further conducted to evaluate the antibacterial effects of Rd at lower concentrations. We found that excellent inhibitory effects were exhibited by 200- and 100-μM Rd with massive bacterial death and serious morphological destruction, and then we investigated the effect on the virulence of *P. gingivalis*, which is mediated by multiple factors. For instance, the long fimbria encoded by the fimA gene plays a critical role in bacterial adherence and coaggregation (Kuboniwa et al., 2009a; Kuboniwa et al., 2009b), the lysine-specific gingipain (kgp) encoded by the kgp gene is responsible for the bacterial proteolytic activity and immune evasion (Kadowaki et al., 2008; Yongqing et al., 2011), while CSH contributes to bacterial adhesion, invasion, and biofilm formation (Li et al., 2003; Baumgarten et al., 2012; Krasowska and Sigler, 2014). According to our data, treatments for *P. gingivalis* with Rd remarkably depressed the expression of virulent genes (*fimA* and *kgp*), as well as decreased CSH, even at a low concentration of 100 μM. Based on this rudimentary knowledge about the antibacterial activities of Rd, we then paid attention to the effect of Rd on *P. gingivalis* biofilms.

In the oral cavity, bacteria always tend to form biofilms, which provide protection and support to the bacteria (O’Toole et al., 2000; Pugliese and Favero, 2002), thus the bacteria can gain better viability, virulence, and antimicrobial resistance than in the planktonic form (Decker et al., 2014; Sweidan et al., 2017). Apart from the inhibitory effects on planktonic *P.gingivalis*, Rd treatments also caused the decrease of the total biomass and the increase of bacterial mortality in *P.gingivalis* biofilms. In contrast, the growth-inhibiting effect on biofilms was only shown by 200-μM Rd, thus further research is warranted to enhance the effect of Rd on persistent growth inhibition. We came to a conclusion that Rd possesses excellent antibacterial and antibiofilm properties towards *P. gingivalis*, along with great attenuation of virulence factors related to bacterial invasion, evasion, and biofilm formation, while the growth-inhibiting effect on the surviving bacteria still needs further investigation.

Facing the challenge from bacteria, inflammatory cytokines such as IL-1β, IL-6 and IL-8 will be released by periodontal tissue cells and immunocytes, and these cytokines play important roles in the process of tissue destruction (Pan et al., 2019; Huang et al., 2021). The anti-inflammatory effect of Rd has been demonstrated in other inflammatory diseases (Liu et al., 2018; Yang et al., 2020) but rarely discussed in periodontitis. HGFs are the most abundant resident cells in periodontal tissues and can continuously produce inflammatory cytokines upon LPS stimulation without LPS tolerance (Ara et al., 2009). Thus, we established an inflammation model by stimulating HGFs with LPS that originated from *P. gingivalis* to investigate the anti-inflammatory effect of Rd. We found that 100-μM Rd potently exerted inhibitory activities towards the production of inflammatory cytokines (IL-1β, IL-6, and IL-8) in HGFs, both in the gene expression and secretion levels. On the other hand, inflammatory cytokines and *P. gingivalis* play a part during the bone resorption process in periodontitis (Zhang et al., 2014; Papathanasiou et al., 2016), which is mediated by promoting osteoclastogenesis (Kitaura et al., 2020). Previous studies demonstrated that protopanaxadiol ginsenosides exert inhibitory efficiency towards osteoclastogenesis (Cheng et al., 2012; He et al., 2012), and our study obtained consistent findings. A total of 100-μM Rd inhibited osteoclastogenesis of not only RAW264.7 cells, a mice preosteoclastic cell line, but also mice BMDMs treated with RANKL and M-CSF, as shown by the dramatic reduction in the amount and volume of TRAP(+) osteoclasts. TRAP is an extracellular enzyme encoded by *Acp5* gene that is commonly utilized to identify osteoclasts, while metalloproteinase (MMP)-9 is vital in mature osteoclasts to mediate bone resorption (Gu et al., 2014; Chen et al., 2021). Nuclear factor of activated T cells (NFAT) c1 is one of the main transcription factors in osteoclastogenesis, which could promote the expression of *Acp5* and *Mmp9* genes (Egusa et al., 2011; Deng et al., 2022). Our results showed that gene expressions of *Nfatc1*, *Mmp9*, and *Acp5* were significantly downregulated by 100-μM Rd as well. These findings attest to the great inhibitory properties of Rd towards periodontal inflammation and osteoclastogenesis *in vitro*.

Based on the positive results as above, the efficacy of Rd was further validated in a mouse periodontitis model, established by ligation and *P. gingivalis* inoculation. It was validated that the antibacterial and anti-inflammatory effects of Rd were consistent with the results *in vitro*, as shown by the decreasing quantity of subgingival *P. gingivalis* and lower levels of inflammatory cytokines (IL-1β and IL-6) in periodontal tissues. Additionally, the alveolar bone resorption and destruction exhibited a dramatic reduction under Rd treatment, despite that, the decrease of TRAP (+) cells did not show statistical significance. We speculated that the discrepancy between osteoclast quantities and bone resorption may have been caused by the limitation of single-layer sections, which could not reveal osteoclasts accurately. In connection with the preceding results, we reasonably inferred that Rd has a prospective capacity to inhibit the growth, virulence, and biofilm of *P. gingivalis*, as well as alleviate local inflammation and bone resorption in periodontitis.

In conclusion, ginsenoside Rd was demonstrated in this study to possess concurrent inhibitory effects on the pathogenicity of *P. gingivalis* and local inflammation and bone resorption, which provided promising evidence for the potential application in the control and prevention of periodontitis.

## Data Availability Statement

The raw data supporting the conclusions of this article will be made available by the authors, without undue reservation.

## Ethics Statement

The animal study was reviewed and approved by the Animal Ethics Committee for Experimental Research of Wuhan University.

## Author Contributions

SZ and YJ designed the experiments. SZ, HG, and ZZ executed the antibacterial experiments *in vitro*. SZ, HY, and ZW executed the anti-inflammation and anti-osteoclastogenesis experiments *in vitro*. SZ, HG, and ZZ executed the experiments *in vivo*. SZ, HY, and ZW analyzed the data. SZ, YJ, and MD wrote the manuscript. YJ and MD made a critical revision. All authors listed have made a substantial, direct, and intellectual contribution to the work and approved it for publication.

## Funding

This work was supported by the National Natural Science Foundation of China (No. 81771084).

## Conflict of Interest

The authors declare that the research was conducted in the absence of any commercial or financial relationships that could be construed as a potential conflict of interest.

## Publisher’s Note

All claims expressed in this article are solely those of the authors and do not necessarily represent those of their affiliated organizations, or those of the publisher, the editors and the reviewers. Any product that may be evaluated in this article, or claim that may be made by its manufacturer, is not guaranteed or endorsed by the publisher.
