# Supplementary material for: Application of Ginsenoside Rd in Periodontitis With Inhibitory Effects on Pathogenicity, Inflammation, and Bone Resorption
Source: Front Cell Infect Microbiol. 2022 Apr 11;12:813953. doi: 10.3389/fcimb.2022.813953 (PMC9035930; doi:10.3389/fcimb.2022.813953)

Supplementary Material

# Supplementary Figures

**
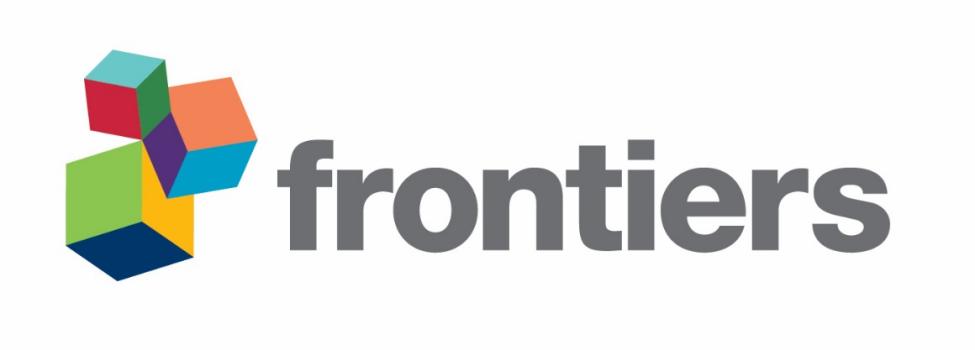
**

**Supplementary Figure 1.** The toxicity of DMSO towards *P.gingivalis*. The results were shown as mean ± SD (n=3). **P* <0.05 versus the normal control.


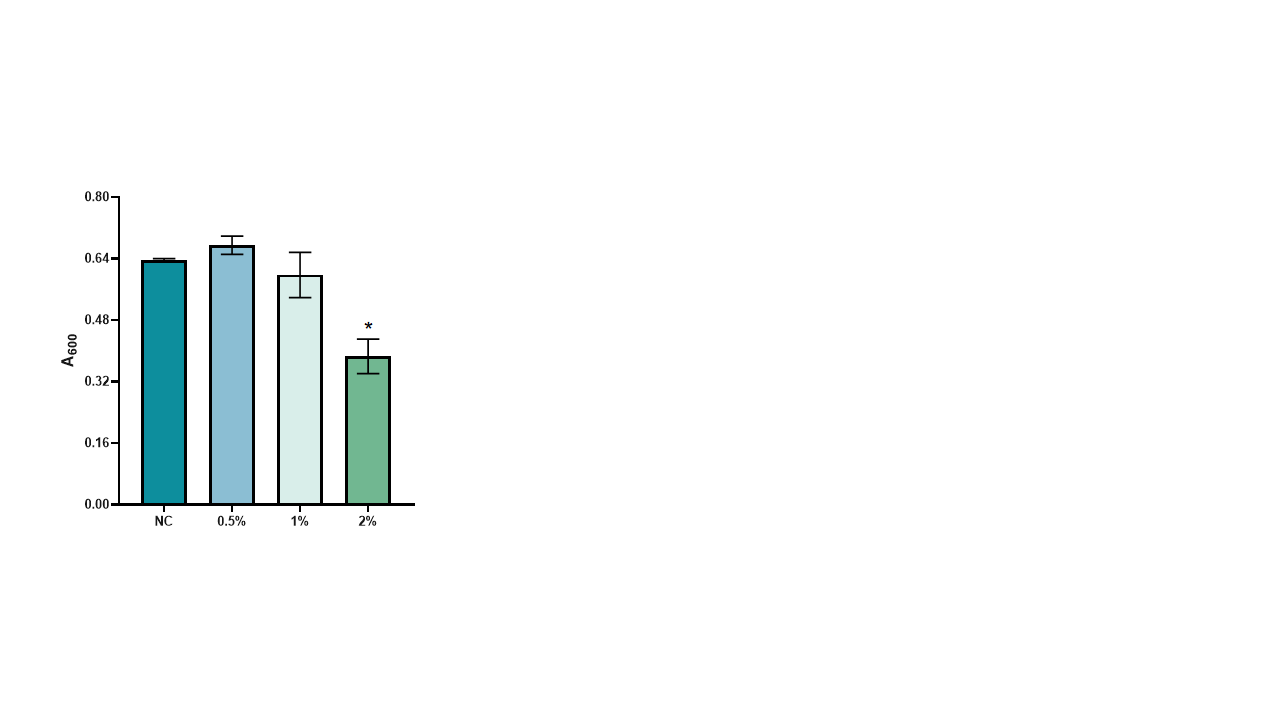


**Supplementary Figure 2.** The cell viability assay for (A)HGFs, (B)RAW264.7 and (C)BMDMs. The results were shown as mean ± SD (n=3). **P* <0.05 versus the normal control.


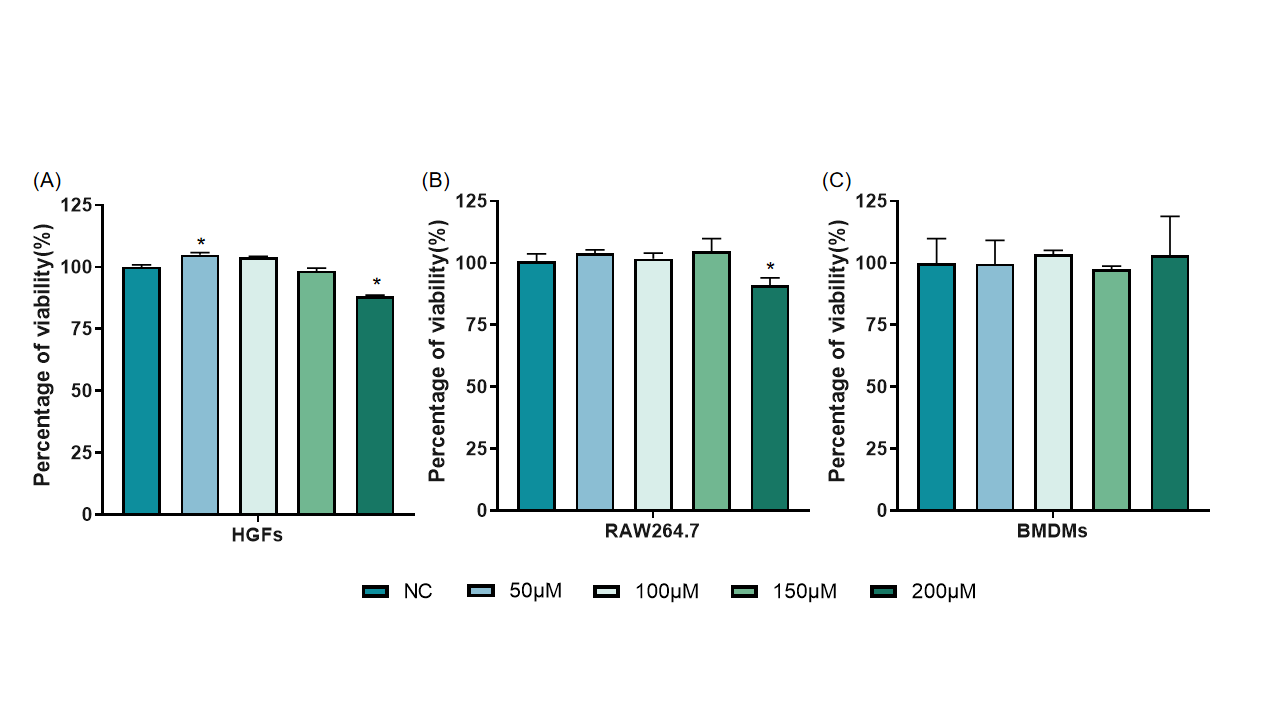


**Supplementary Figure 3.** The biological security of Rd. (A)Weight records of mice. The results were shown as mean ± SD (n=3). **P* <0.05 versus the normal control. (B)HE staining for the heart, liver, spleen, lung, kidney of mice. Photographs were taken under 20× magnification.
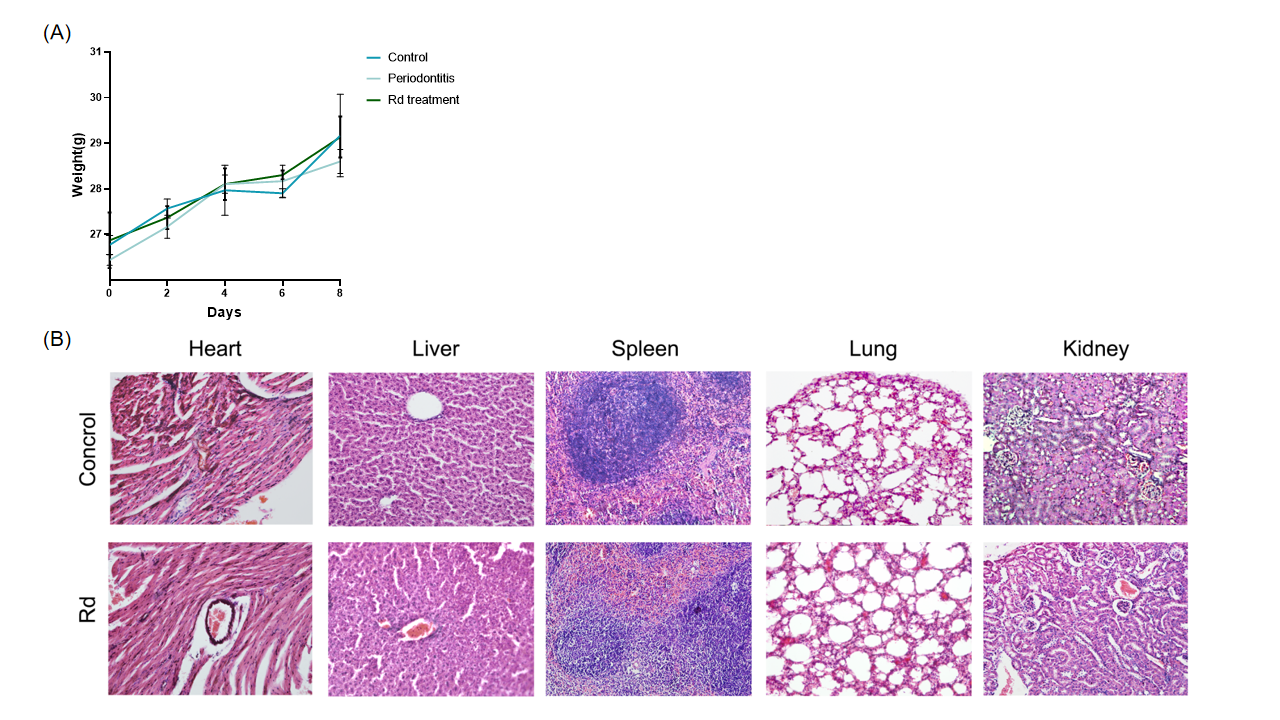


**Supplementary Figure 4.** Photographs of mice alveolar bones were taken under 3.2× magnification. The CEJ-ABC distances were indicated by the white lines and were measured according to the scale. The results were shown as mean ± SD (n=3). **P* <0.05 versus the normal control. #*P* <0.05 versus the periodontitis group.


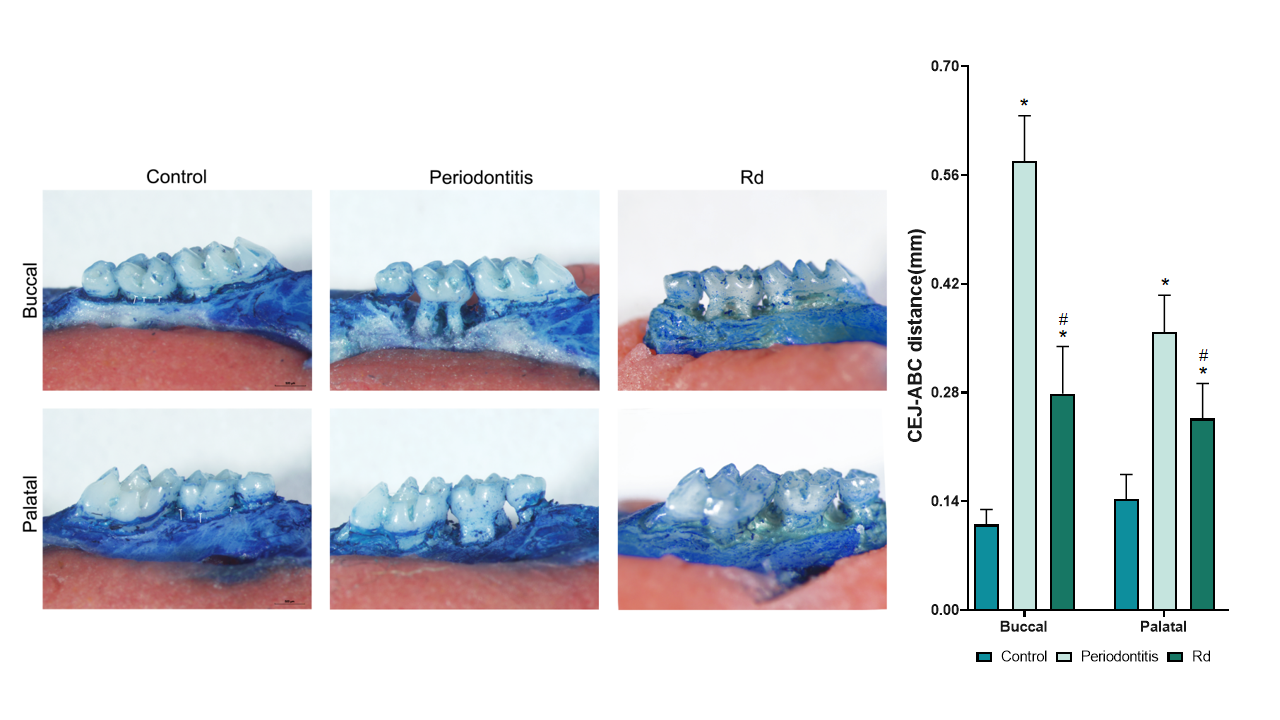

Supplement: Supplementary file 1 [file DataSheet_1.docx]
